# Ileocecal ulcers accompanied by relapsing polychondritis: a case report

**DOI:** 10.1186/2193-1801-3-714

**Published:** 2014-12-07

**Authors:** Yoko Kawakami, Katsuya Endo, Tomonori Ishii, Sho Haneda, Fumiyoshi Fujishima, Yoichi Kakuta, Hisashi Shiga, Yoshitaka Kinouchi, Tooru Shimosegawa

**Affiliations:** Division of Gastroenterology, Department of Internal Medicine, Tohoku University Graduate School of Medicine, 1-1 Seiryo, Aoba-ku, Sendai, 980-8574 Japan; Department of Hematology and Rheumatology, Tohoku University Graduate School of Medicine, 1-1 Seiryo, Aoba-ku, Sendai, 980-8574 Japan; Division of Gastrointestinal Surgery, Department of Surgery, Tohoku University Hospital, 1-1 Seiryo, Aoba-ku, Sendai, 980-8574 Japan; Department of Pathology, Tohoku University Hospital, 1-1 Seiryo, Aoba-ku, Sendai, 980-8574 Japan

**Keywords:** Intestinal Behçet’s disease, Relapsing polychondritis, Ileocecal ulcers, MAGIC syndrome

## Abstract

**Introduction:**

Mouth and genital ulcers with inflamed cartilage (MAGIC) syndrome is a rare overlap syndrome that includes features characteristic of both Behçet’s disease (BD) and relapsing polychondritis (RP).

**Case description:**

A 30-year-old female complained of lower abdominal pain and bloody stools during medical treatment for RP. Total colonoscopy revealed oval-shaped deep ulcers on the terminal ileum similar to those of intestinal BD. After performing the ileocecal resection, both RP and gastrointestinal lesions relapsed, but improved with infliximab treatment.

**Discussion and evaluation:**

During medical treatment for RP, we experienced a rare case with ileocecal ulcers similar to intestinal BD. Although our case did not meet the diagnosis criteria of intestinal BD because of the lack of BD’s major clinical symptoms, intestinal lesions shared quite similar features with intestinal BD. Our case could possibly be a rare subtype of MAGIC syndrome that had the features characteristic of both intestinal BD and RP.

**Conclusions:**

We described a rare case of ileocecal ulcers without any BD symptoms but accompanied by RP, possibly be a subtype of MAGIC syndrome.

**Electronic supplementary material:**

The online version of this article (doi:10.1186/2193-1801-3-714) contains supplementary material, which is available to authorized users.

## Background

Behçet’s disease (BD) is a chronic relapsing inflammatory disease with multiorgan system involvement which is clinically characterized by oral aphthae, genital ulcers, cutaneous lesions, and ophthalmologic manifestations. BD patients are common in Asia, the Middle East and the Mediterranean regions; however, it is uncommon in Western countries (Sakane et al. 1999). It is reported that 3–16% of the patients with BD have gastrointestinal (GI) tract involvement (Hisamatsu et al. 2014). BD patients with GI lesions are subcategorized as intestinal BD because these patients often have severe GI complications and a poor prognosis. In typical cases of intestinal BD, patients often develop an oval-shaped deep ulcer in the ileoceacal lesion which sometimes causes massive bleeding and perforation. Recently, the consensus statements for the diagnosis and management for intestinal BD has been proposed from the Japanese Committee of Experts (Hisamatsu et al. 2014; Kobayashi et al. 2007). In this statement, diagnostic criteria and therapeutic strategy are mentioned. According to the statement, besides GI lesions, BD symptoms such as mouth, eye, skin, and genital lesions are necessary for the diagnosis of intestinal BD (Kobayashi et al. 2007).

Relapsing polychondritis (RP) is a rare and chronic disease characterized by recurrent inflammation episodes of the cartilaginous tissue and other proteoglycanrich tissues (McAdam et al. 1976; Damiani and Levine 1979). Patients with RP have diverse symptoms in the auricle, nose, eyes, tracheal cartilage, joints, heart, and blood vessels. The etiology of RP is unknown, but an autoimmune origin has been hypothesized. Approximately 30% of the cases are associated with other auto-immune diseases (McAdam et al. 1976). Criteria for diagnosis, suggested by McAdam et al. (1976), include three or more of the following clinical features: 1) bilateral auricular chondritis, 2) nonerosive seronegative inflammatory polyarthritis, 3) nasal chondritis, 4) ocular inflammation, 5) respiratory tract chondritis, and 6) audiovestibular damage, with compatible histological features in a cartilage biopsy specimen.

Mouth and genital ulcers with inflamed cartilage (MAGIC) syndrome is a rare overlap syndrome that includes features characteristic of both BD and RP (Firestein et al. 1985). Although there have been no established diagnosis criteria, both mucosal symptoms (oral aphthae or genital ulcers) and cartilage inflammation are thought to be necessary for the diagnosis of MAGIC syndrome as Firestein et al. initially described in 1985 (Firestein et al. 1985). Most of the cases reported as MAGIC syndrome had no GI lesions. However, a few cases of MAGIC syndrome with GI lesions besides mucosal lesions and cartilage inflammation (Firestein et al. 1985; Imai et al. 1997; Kotter et al. 2006; Minami et al. 2009) have been reported. Until now, there has been no other case report of RP with typical Behçet’s GI lesions without any mucosal lesions.

During medical treatment for RP, we experienced a rare case with ileocecal ulcers similar to intestinal BD. Although our case did not meet the diagnosis criteria of intestinal BD because of the lack of BD’s major clinical symptoms, intestinal lesions shared quite similar features with intestinal BD. Our case could possibly be a rare subtype of MAGIC syndrome that had the features characteristic of both intestinal BD and RP. In this report, we have reported the case precisely and made a discussion focused on the diagnosis and treatment with literature review.

## Case presentation

**Figure 1:**
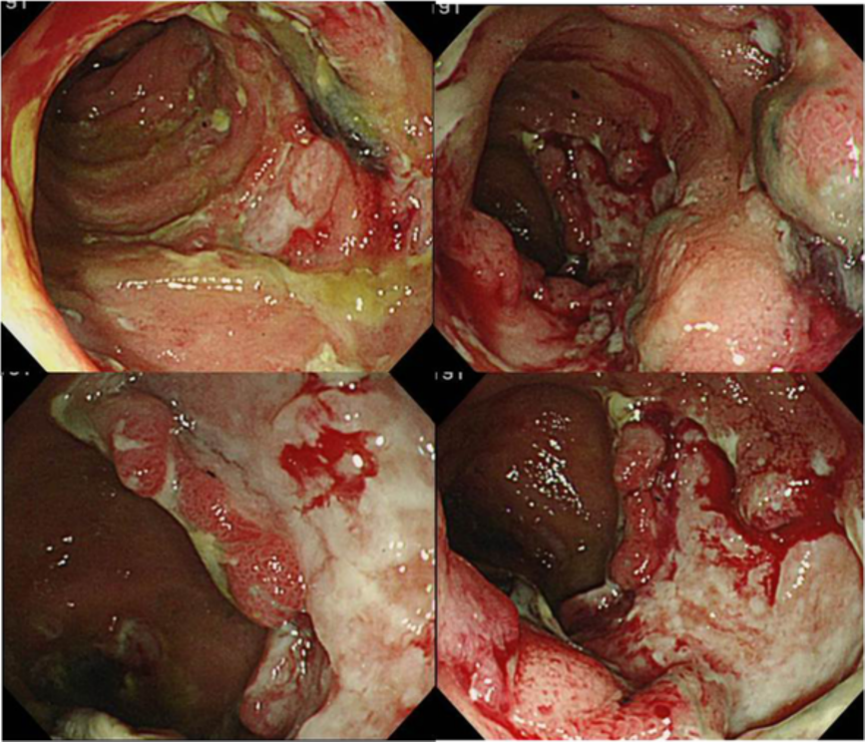
**Total colonoscopy (TCS) revealed oval-shaped deep ulcers on the terminal ileum with deformity and destruction of the ileocecal valve.**

**Figure 2:**
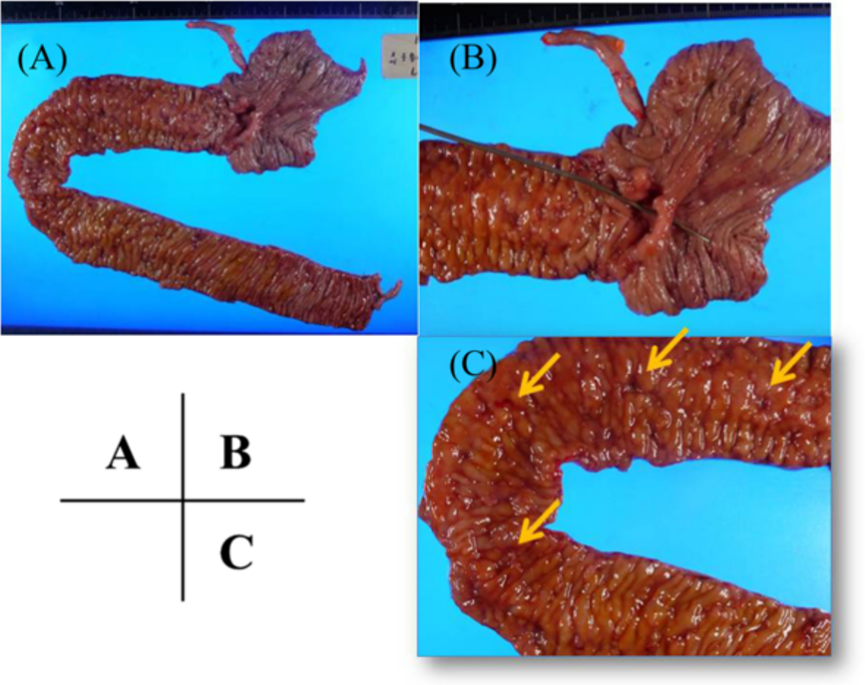
**Gross findings from the resected specimen. (A and B)** The ileocecal valve was highly deformed and destroyed by deep ulcers. **(C)** Large and small ulcers were diffusely-scattered on the ileal mucosa, from the ileocecal valve to 30 cm the oral side.

**Figure 3:**
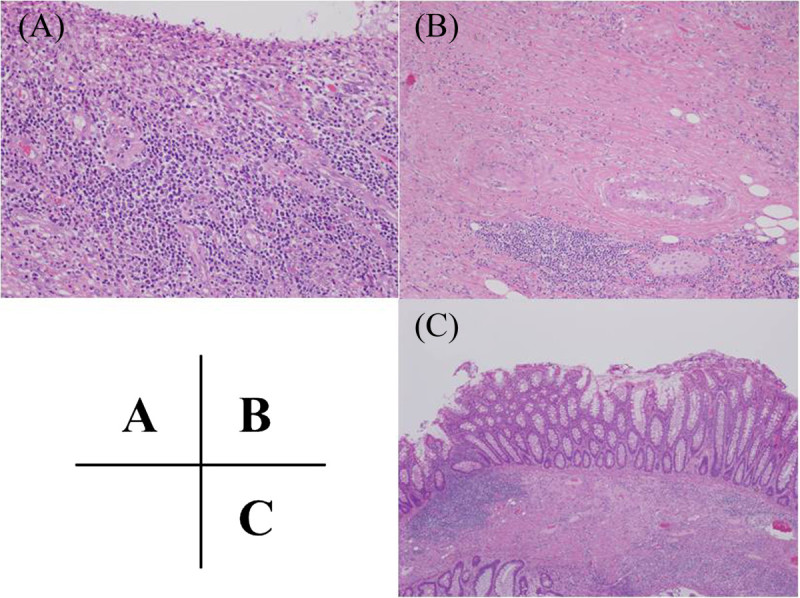
**Pathological findings from the resected specimen. (A and B)** There was nonspecific inflammation in the ulcers. There were no specific findings such as granulomas, CMV-infected cells, vasculitis or thrombus formation. **(C)** The mucosa around the ulcers remained relatively normal in structure. (Hematoxylin and eosin stain).

A 30-year-old female with a fever, scleritis, and auricular pain for a duration of 3 months was examined. She was finally diagnosed with RP from her symptoms, auricle biopsy and response to corticosteroid therapy according to the proposed diagnostic criteria (McAdam et al. 1976; Damiani and Levine 1979). She frequently took non-steroidal anti-inflammatory drugs (NSAIDs) along with predonisolone for pain control. Subsequently, she experienced episodes of lower abdominal pain 2 months after the initial diagnosis of RP. Although her abdominal symptoms temporally subsided with steroid pulse therapy, the abdominal pain relapsed and the bloody stools as well as anemia emerged after terminating steroid pulse therapy. As a result, we started to investigate her abdomen and GI tract. Laboratory tests revealed an increased inflammatory response (white blood cells 10,300/μl; C-reactive protein, 5.4 mg/dl), hypoalbuminemia (total protein, 5.9 g/dl, albumin, 2.9 g/dl), and anemia (red blood cells, 3.20 × 106/μl, hemoglobin, 8.7 g/dl, hematocrit, 27.2%) (Table 1). Abdominal contrast-enhanced computed tomography (CT) scan revealed thickening of the ileocecal wall, and positron emission tomogaraphy (PET) revealed accumulation in the intestinal tract at the terminal ileum. Total colonoscopy (TCS) revealed oval-shaped deep ulcers on the terminal ileum with deformity and destruction of the ileocecal valve (Figure 1). In addition, reddish edematous mucosa and ulcers were detected in the oral side of the ileum. Biopsy specimens taken from the terminal ileum showed nonspecific inflammatory findings. There were no abnormal findings in her large intestinal mucosa and upper GI tracts. We highly suspected the possibility of intestinal BD from the characteristic endoscopic findings, but the case did not meet the diagnostic criteria because of lacking the major BD symptoms such as oral aphthoid ulcers, eye symptoms, skin lesions or genital ulcers. In addition, other diseases such as NSAIDs-induced enteritis were not completely denied; therefore, we selected a conservative therapy and planned a close follow-up. After restricting food and NSAIDs from the initial TCS, slight improvements were observed in her abdominal symptoms. However, no significant change was evident on colonoscopy performed 4 weeks after the initial TCS. Differential diagnoses included intestinal BD, Crohn’s disease, NSAIDs-induced enteritis, malignant lymphoma, and infectious colitis such as cytomegalovirus colitis or enteric tuberculosis. However, the test results, including a biopsy, culture, and various imaging tests did not provide a definitive diagnosis. We decided to perform surgery in order to make a definite diagnosis and avoid the GI perforation because of the presence of deep and active ulcers. An ileocecal resection was performed 5 weeks after the initial TCS. Gross findings of the resected specimen showed that the ileocecal valve was highly deformed and destroyed by deep ulcers. Furthermore, large and small ulcers were diffusely-scattered on the ileal mucosa, from the ileocecal valve to 30 cm on the oral side (Figure 2). Histological examination revealed ulcer formation with destruction of muscularis propria. The ulcer base was rather flat and showing involvement of nonspecific chronic inflammation with fibrotic change. The mucosa around the ulcers remained relatively normal in structure (Figure 3). There were no specific findings such as granulomas, CMV-infected cells, vasculitis or thrombus formation; thus, we remained to believe intestinal BD as the diagnosis.

**Table 1:** **Laboratory data on the first visit**

Peripheral blood		Blood chemistry	
WBC	10300/μl	AST	27 IU/l
RBC	3.2 × 106/μl	ALT	27 IU/l
Hb	8.7 g/dl	ALP	199 IU/l
Hct	27.2%	Γ-GTP	20 IU/l
MCV	85.1 fl	LDH	131 IU/l
MCH	27.1 pg	T-Bil	0.3 mg/dl
MCHC	31.8%	CPK	8 IU/l
PLT	47.7 × 104/μl	TP	5.9 g/dl
		Alb	2.9 g/dl
**Serological test**		BUN	14 mg/dl
CRP	5.4 mg/dl	Cr	0.5 mg/dl
		Na	138 mEq/l
		K	4.3 mEq/l
		Cl	100 mEq/l
		Ca	9 mg/dl

*WBC* white bold cell, *RBC* red blood cell, *Hb* hemoglobin, *Hct* hematocrit, *MCV* mean corpuscular volume, *MCH* mean corpuscular volume, *MCHC* mean corpuscular hemoglobin concentration, *Pl* tplatelets, *AST* aspartate aminotransferese, *ALT* alanineaminotransferase, *ALP* alkaline phosphatase, *γ-GTP* γ-glutamyltransferase, *LDH* lactate dehydrogenase, *T-Bil* total bilirubin, *CPK* ccreatine phosphokinase, *TP* total protein, *Alb* albumin, *BUN* blood urea nitrogen, *Cr* creatinine, *Na* sodium, *K* potassium, *Cl* chloride, *Ca* calcium, *CRP* C-reactive protein.

**Figure 4:**
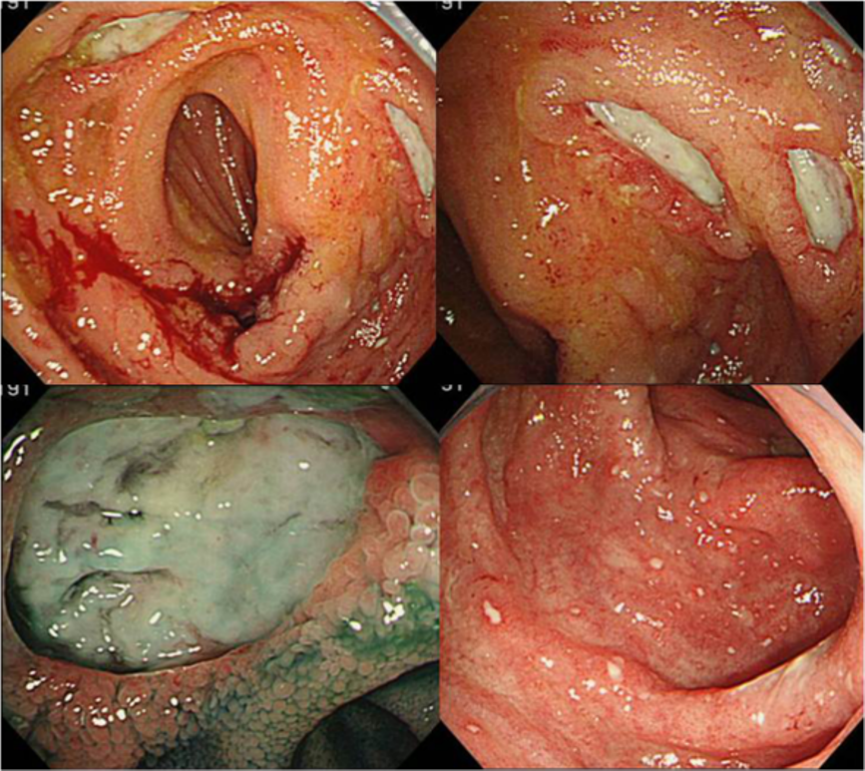
**Total colonoscopy revealed multiple deep and round ulcers developing on the anastomotic site, and multiple erosions and aphthae were observed on the oral side of the small and large intestine.**

**Figure 5:**
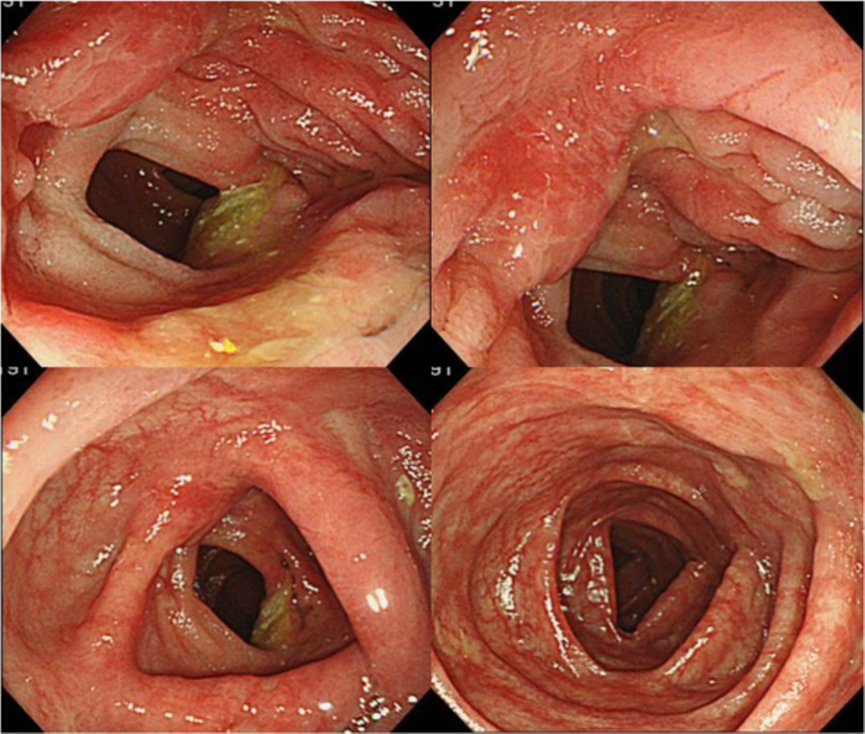
**Total colonoscopy shows significant improvement of the ulcers on the anastomosis site.**

After the operation, she was in stable condition. We followed her progress on an outpatient basis while tapering her prednisolone dosage. However, RP symptoms such as fever, scleritis, and auricular pain relapsed 5 months after the operation, in addition to the recurrence of abdominal pain and watery diarrhea. This led us to perform TCS which revealed multiple deep and round ulcers developing at the anastomotic site. We also observed multiple erosions and aphthae on the oral side of the small intestine and large bowel from the ascending colon to the rectum (Figure 4). We initiated infliximab (IFX) administration, which has been reported to be effective against both RP and BD. After the administration of IFX, her cartilaginous and abdominal symptoms dramatically improved. A follow-up TCS was performed at the end of the fourth administration of IFX. We identified ulcer scars at the anastomosis site and inflammation of the large intestine improved (Figure 5). We recognized that both RP and BD were responsive to IFX administration. Currently as an outpatient, she is regularly receiving IFX treatment and continues to remain in good health.

## Discussion

Intestinal BD is a subcategory of BD that develops characteristic inflammation in the GI tracts. Although some cases involve the esophagus or small intestine (Hisamatsu et al. 2014), patients often have an oval-shaped deep ulcer in the ileocecal lesion. Patients with intestinal BD have symptoms such as abodominal pain, bloody stools and diarrhea. Sometimes massive bleeding or perforation can occur which leads to a poor prognosis. Our patient also had lower abdominal pain and bloody stool during RP treatment that were consistent with intestinal BD symptoms. As for the diagnosis of intestinal BD, Japanese expert’s committee proposed the diagnosis criteria initially in 2007 (Kobayashi et al. 2007). Recently, they published the 2^nd^ edition of this statement (Hisamatsu et al. 2014). According to their statement, the diagnosis of intestinal BD could be made if the patient meets the following criteria: A) There is a typical oval-shaped large ulcer in the terminal ileum, and clinical findings meet the diagnostic criteria of BD or, B) There are ulcerations or inflammation in the small or large intestine, and clinical findings meet the diagnostic criteria of BD. Furthermore, acute appendicitis, infectious enteritis, tuberculosis, Crohn’s disease, nonspecific colitis, drug-associated colitis and other diseases that mimic intestinal BD should be excluded by clinical findings, radiology, and endoscopy. Our case developed deep oval-shaped ulcers in the terminal ileum resembling those of intestinal BD. Gross findings of the resected specimen showed that ileocecal valve was highly deformed and destroyed by deep ulcers. Histological examination revealed that nonspecific inflammation and ulcers had reached the muscularis propria. There were no specific findings such as granulomas, CMV-infected cells, vasculitis or thrombus formation. These GI findings itself were quite similar to those of intestinal BD. However, we could not confirm a diagnosis of intestinal BD because of the lack of oral aphthoid ulcers, eye symptoms, skin lesions or genital ulcers which were necessary for the diagnosis. We believed there was a high possibility that the present case could be related to intestinal BD. The disease concept of “Simple ulcer”, which is defined as deep ileocecal ulcers difficult to distinguish from intestinal BD but lacks the other clinical symptoms of BD, has been advocated in Japan (Murano et al. 2011). Although it is an inconclusive discussion, the simple ulcer has been considered to be related to intestinal BD.

RP is a rare and chronic disease characterized by recurrent inflammation episodes of cartilaginous tissue and other proteoglycan-rich tissues. RP is a multisystem autoimmune disease and can coexist with a systemic vasculitis. Although some cases of RP accompanied by ulcerative colitis was reported (Firestein et al. 1985; Benito Calavia et al. 1997; Kawano et al. 2001), GI symptoms are uncommon in RP (Firestein et al. 1985) and there have been few reports of intestinal BD with RP. The criteria for the diagnosis of RP were proposed by McAdam et al. (1976), which include three or more of the following clinical features: 1) bilateral auricular chondritis, 2) nonerosive seronegative inflammatory polyarthritis, 3) nasal chondritis, 4) ocular inflammation, 5) respiratory tract chondritis, and 6) audiovestibular damage, with compatible histological features in a cartilage biopsy specimen. These criteria were slightly expanded by Damiani and Levine (1979) to include reaction to treatment with corticosteroids or dapsone (Damiani and Levine 1979). Our patient had scleritis, auricular pain, and vertigo. Because these clinical findings, auricle biopsy results and the response to corticosteroid fulfilled the above diagnostic criteria, we were able to confirm our diagnosis of RP.

MAGIC syndrome was initially described by Firestein et al. (1985). MAGIC syndrome is defined as an overlap syndrome that includes features characteristic of both BD and RP. Although a few authors suggest that MAGIC syndrome is not a disease entity but merely the association of BD with polychondritis (Kotter et al. 2006; Kotter 2006), many authors support the concept of this syndrome (Imai et al. 1997; Orme et al. 1990; Kim et al. 2005; Caceres et al. 2006; Nanke et al. 2006; Hidalgo-Tenorio et al. 2008; Mekinian et al. 2009; Geissal and Wernick 2010; Gertner 2004). They suggest that the similarities in the clinical manifestations and pathological findings in BD and RP lead a common pathogenetic pathway, so that there is a close relationship between both diseases (Orme et al. 1990; Kim et al. 2005; Hidalgo-Tenorio et al. 2008; Mekinian et al. 2009; Gertner 2004).

The definite diagnosis criteria of MAGIC syndrome have not been established. However, both mucosal symptoms (oral aphthae or genital ulcers) and cartilage inflammation are thought to be necessary for diagnosis according to the first descriptions by Firestein et al. (1985). It is a very uncommon disorder, and until now, only 24 cases have been reported (Firestein et al. 1985; Imai et al. 1997; Kotter et al. 2006; Minami et al. 2009; Orme et al. 1990; Kim et al. 2005; Caceres et al. 2006; Nanke et al. 2006; Hidalgo-Tenorio et al. 2008; Mekinian et al. 2009; Geissal and Wernick 2010; Gertner 2004; Le Thi et al. 1993; Fernandez-Monras et al. 1997; Ng et al. 2007). All of the 24 cases had both mucosal symptoms and cartilage inflammation, and met the Firestein’s definition. Five out of the 24 cases were reported to have colitis or ileocecal ulcers (Firestein et al. 1985; Imai et al. 1997; Kotter et al. 2006; Minami et al. 2009). In these 5 cases, 2 cases had ileocecal ulcers that were thought to be a typical GI lesion of intestinal BD (Table 2). Therefore, patients with MAGIC syndrome who have characteristic ileoceacal ulcers consistent with intestinal BD are rare, but do exist. Until now, there has been few report of a RP with intestinal BD that had no mucosal lesions. Our patient did not present symptoms such as oral and genital ulcers, but had ileocecal ulcers consistent with intestinal BD during the treatment for RP. Our case completely met the diagnostic criteria for RP, and had a typical GI lesion similar to intestinal BD. However, the case did not completely meet the MAGIC syndrome definition, because she had no mucosal lesions. It remains possible that the using corticosteroid for RP might cover her mucosal symptoms. So we consider that our case could possibly be a rare subtype of MAGIC syndrome that had the complete features of RP and partial characteristics of intestinal BD.

**Table 2:** **Summary of the cases of MAGIC syndrome with GI lesions**

Case no.	Author	Age/gender	Clinical features					
			***Chondritis***	***Chondritis***	***Genital lcers***	***Ocular inflammation***	***Skin manifestation***	***Gastrointestinal***
1	Firestein et al. (1985)	59, M	Auricular chondritis	(+)	(+)	(−)	(−)	Duodenal ulcer, intestinal fistula
2	Imai et al. (1997)	39, F	Auricular chondritis	(+)	(+)	Keratoiritis	Erythema nodosum	Colitis
3	Kotter et al. (2006)	59, M	Auricular chondritis	(+)	(−)	Scleritis	(−)	Aphthous colitis
4	Minami et al. (2009)	30, M	Auricular chondritis,	(−)	(+)	Scleritis	(−)	Ileocecal ulcer
5	Our case	30, F	Auricular chondritis, nasal chondritis	(−)	(−)	Scleritis	(−)	Ileocecal ulcer

In conventional therapy for intestinal BD, 5-aminosalicylic acid (5-ASA) systemic corticosteroid, and immunosuppressive agents have been used (Hisamatsu et al. 2014). However, many patients become refractory to these drugs. Recently, the efficacy of anti-tumor necrosis factor (TNF)α antibodies, such as infliximab and adalimumab was reported (Sfikakis et al. 2001; Travis et al. 2001; Hassard et al. 2001; Kram et al. 2003; Ju et al. 2007; Byeon et al. 2007; Naganuma et al. 2008; Iwata et al. 2011; Kinoshita et al. 2013). The 2^nd^ edition of the consensus statement of the Japanese Experts Committee described that infliximab and adalimumab should be considered as a standard therapy for intestinal BD (Hisamatsu et al. 2014). As for the treatment for RP, various drugs including corticosteroid, NSAIDs, colchicine, hydroxychloroquine, dapsone, methotrexate, azathioprine, cyclophoshamide, and cyclosporine have been used (Kemta Lekpa et al. 2012). In many cases, corticosteroid can be effective; however, not all patients respond adequately. In recent years, several patients were reported to be treated with infliximab successfully (Kemta Lekpa et al. 2012). Pharmacological treatment options for MAGIC syndrome are comprised of NSAIDs, colchicines, corticosteroids, immunosuppressants, and biologics, but there is no specific treatment. To date, only three cases of MAGIC syndrome treated with anti-TNFα have been reported (Hidalgo-Tenorio et al. 2008; Mekinian et al. 2009; Geissal and Wernick 2010). They were all treated with IFX (3 ~ 5 mg/kg) and two of the cases have continued to be in remission.

Our case was corticosteroid dependent and refractory. Although both RP symptoms and GI symptoms improved during steroid pulse therapy before the operation, the symptoms relapsed after stopping the therapy. Furthermore, after the ileocecal resection, both RP and GI lesions relapsed at the same time when reducing the prednisolone dosage. As mentioned above, infliximab has been reported to be effective to RP, intestinal BD and MAGIC syndrome. As a result, we administrated infliximab in order to avoid the continuous high dose usage of steroids. The patient dramatically demonstrated good response to infliximab. Examples of administering biologic therapies such as infliximab to treat patients with intestinal BD, RP, and MAGIC syndrome have gradually increased (Hisamatsu et al. 2014; Kobayashi et al. 2007; Hidalgo-Tenorio et al. 2008; Mekinian et al. 2009; Geissal and Wernick 2010; Kemta Lekpa et al. 2012; Hatemi et al. 2012). Considering the good response to infliximab in other cases and ours, TNFα may play a fundamental role in MAGIC syndorome. Further investigations are necessary to establish the appropriate therapeutic strategy for MAGIC syndrome.

## Conclusions

We described a rare case of ileocecal ulcers without any BD symptoms but accompanied by RP, possibly be a subtype of MAGIC syndrome. Additional cases and further investigation are required to clarify the pathogenesis of this rare syndrome.

## Consent

Written informed consent was obtained from the patient for the publication of this report and any accompanying images.

## Authors’ original submitted files for images

Below are the links to the authors’ original submitted files for images. Authors’ original file for figure 1 Authors’ original file for figure 2 Authors’ original file for figure 3 Authors’ original file for figure 4 Authors’ original file for figure 5 Authors’ original file for figure 6 Authors’ original file for figure 7
