# Supplementary figures and images for: Ileocecal ulcers accompanied by relapsing polychondritis: a case report
Source: Springerplus. 2014 Dec 7;3:714. doi: 10.1186/2193-1801-3-714 (PMC4320181; doi:10.1186/2193-1801-3-714)

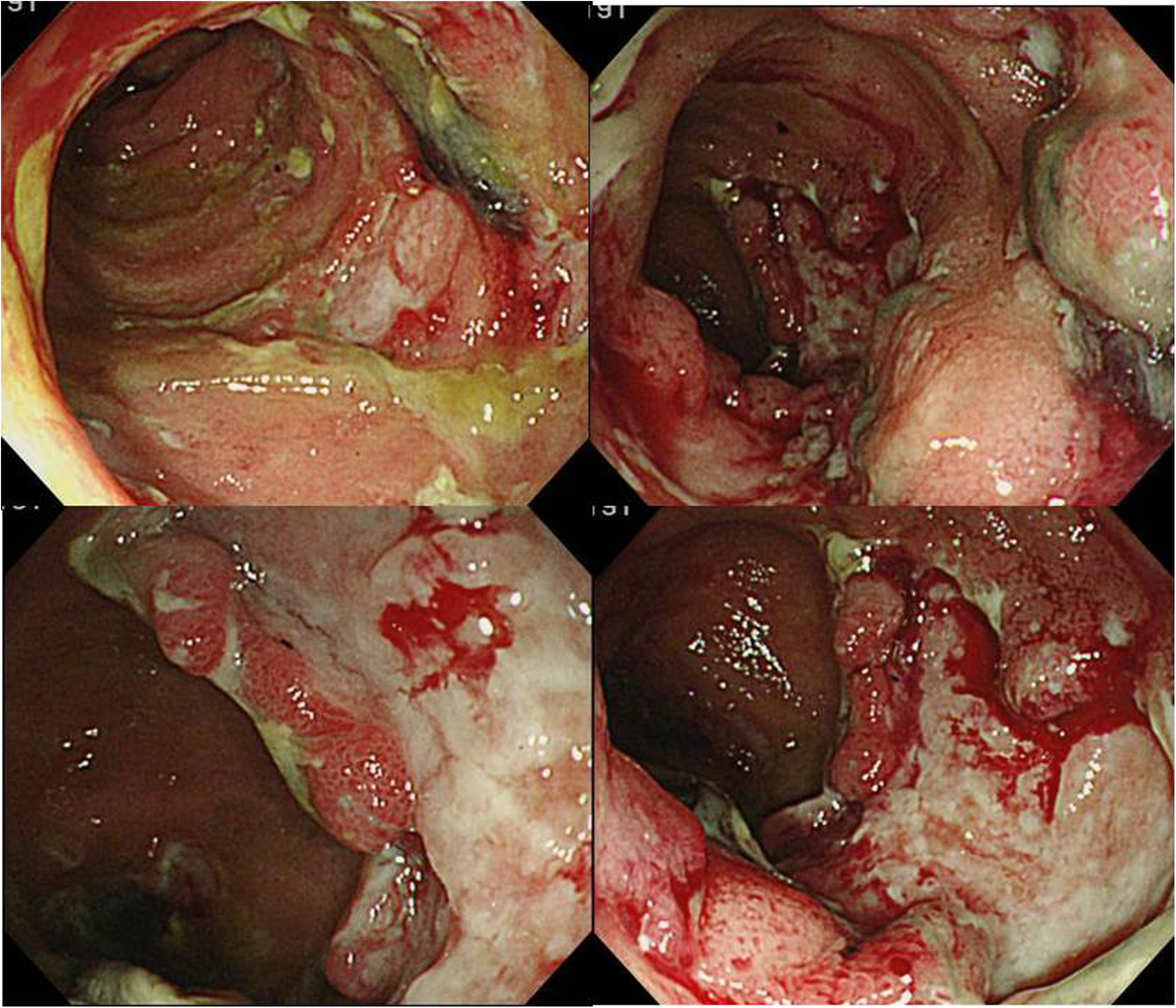

Supplement: Supplementary file 1 — Authors’ original file for figure 1 [file 40064_2014_1482_MOESM1_ESM.tif]

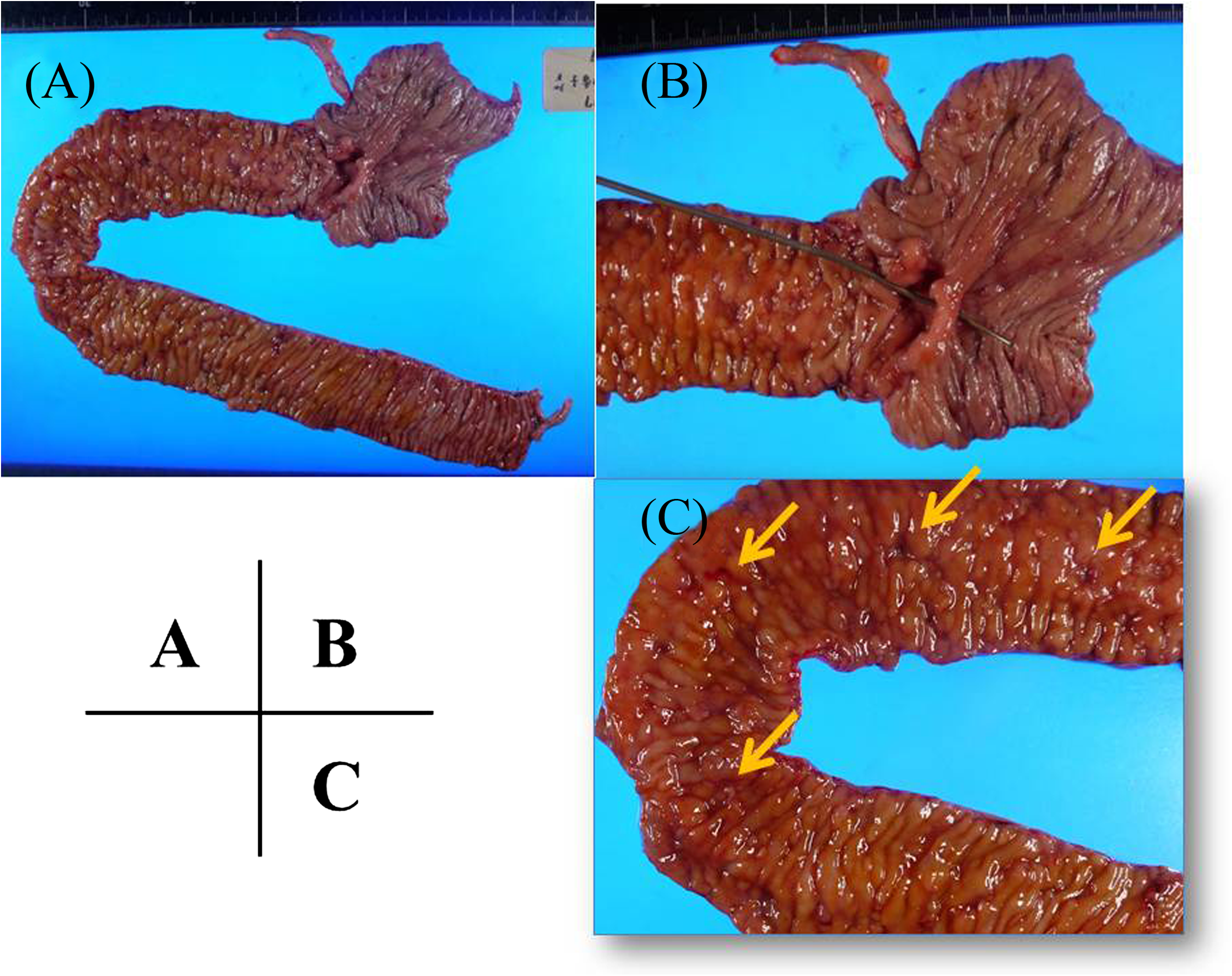

Supplement: Supplementary file 2 — Authors’ original file for figure 2 [file 40064_2014_1482_MOESM2_ESM.tif]

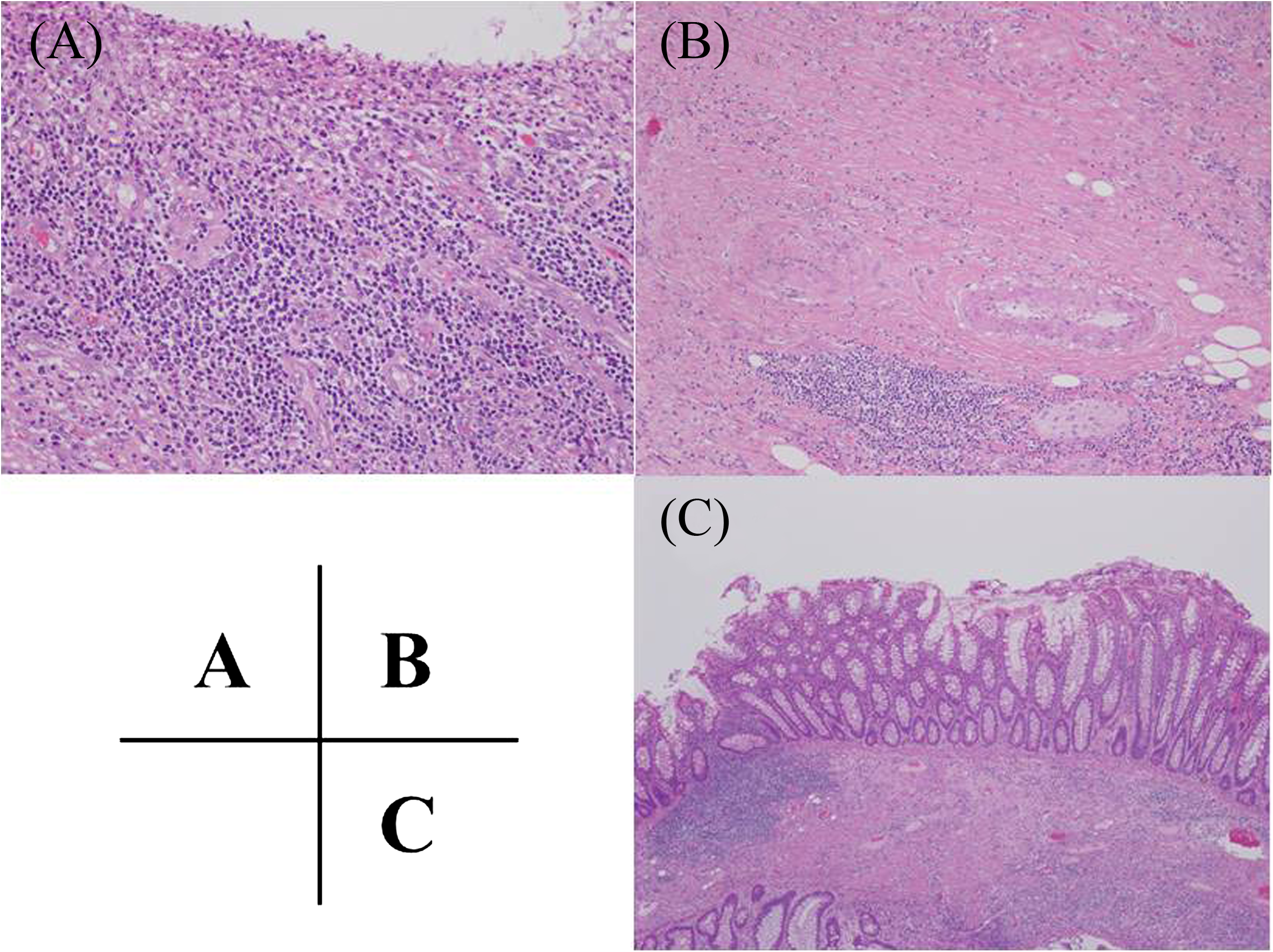

Supplement: Supplementary file 3 — Authors’ original file for figure 3 [file 40064_2014_1482_MOESM3_ESM.tif]

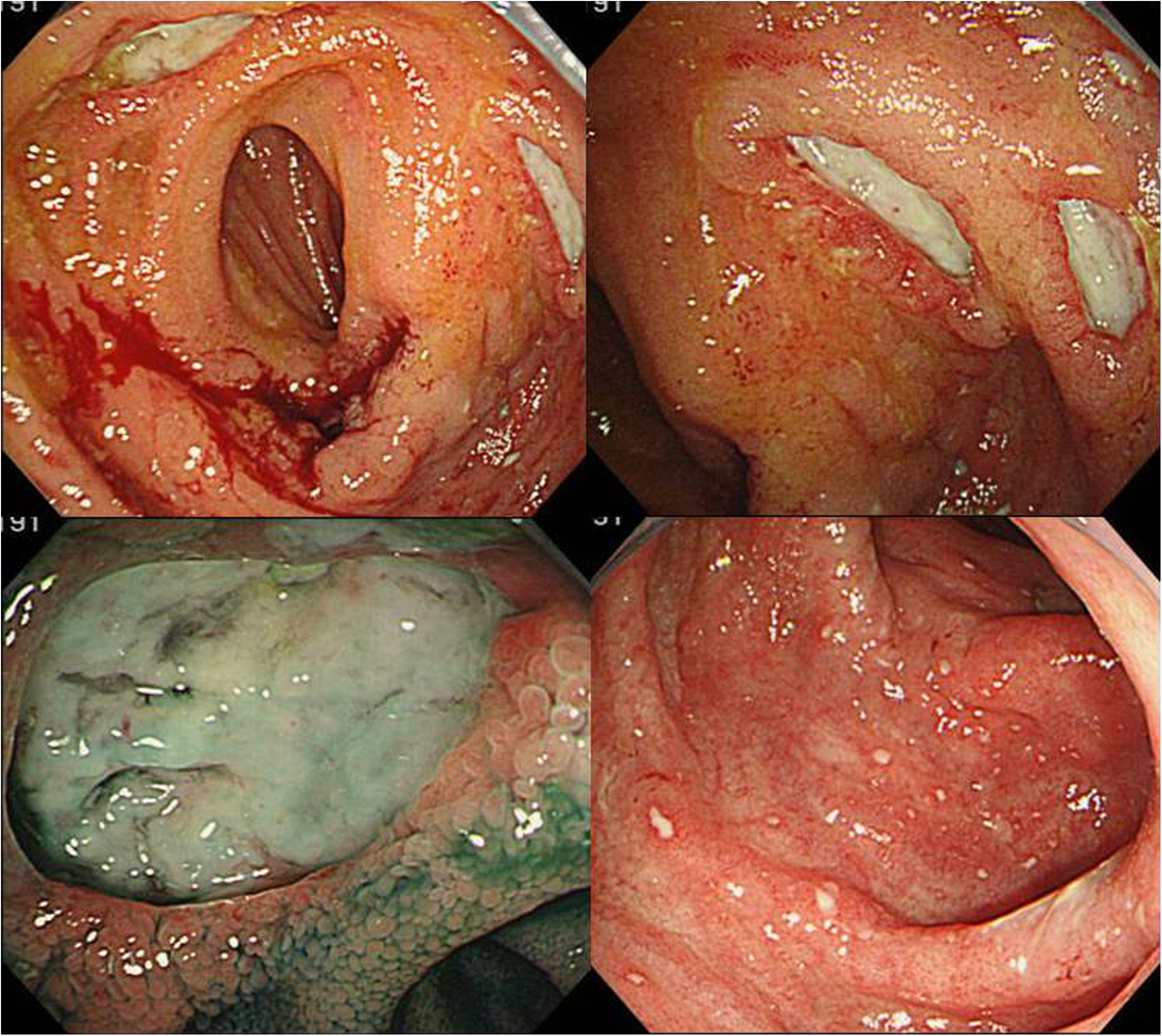

Supplement: Supplementary file 4 — Authors’ original file for figure 4 [file 40064_2014_1482_MOESM4_ESM.tif]

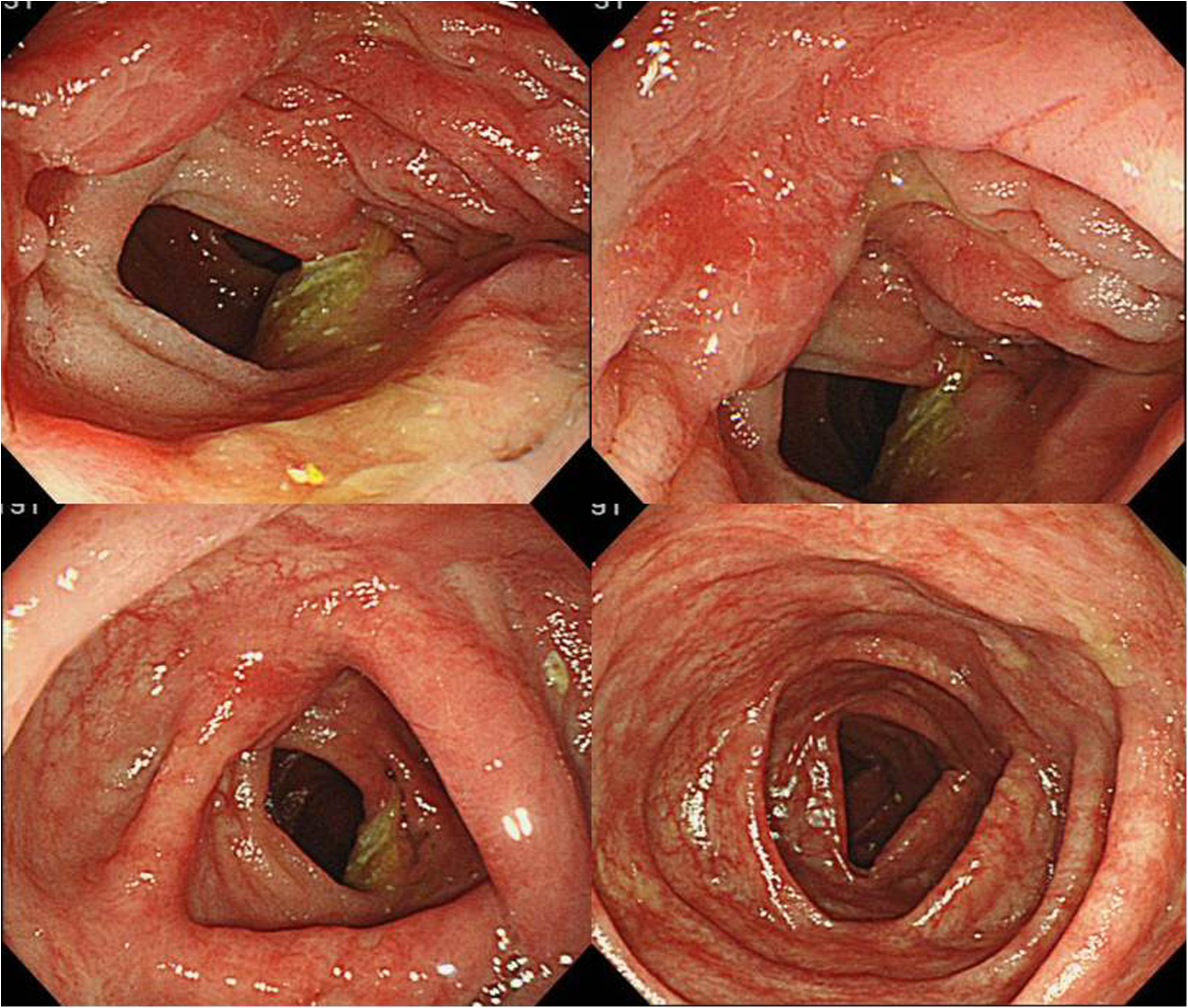

Supplement: Supplementary file 5 — Authors’ original file for figure 5 [file 40064_2014_1482_MOESM5_ESM.tif]
