# Supplementary material for: Ileocecal ulcers accompanied by relapsing polychondritis: a case report
Source: Springerplus. 2014 Dec 7;3:714. doi: 10.1186/2193-1801-3-714 (PMC4320181; doi:10.1186/2193-1801-3-714)
Supplement: Supplementary file 6 — Authors’ original file for figure 6 [file 40064_2014_1482_MOESM6_ESM.pdf]

Table1 .Laboratory Data on the first visit

Peripher			Blood		
WBC	10300	/μl	AST	27	IU/l
RBC	3.2	$\times 10^6$/μl	ALT	27	IU/l
Hb	8.7	g/dl	ALP	199	IU/l
Hct	27.2	%	Γ-GTP	20	IU/l
MCV	85.1	fl	LDH	131	IU/l
MCH	27.1	pg	T-Bil	0.3	mg/dl
MCHC	31.8	%	CPK	8	IU/l
PLT	47.7	$\times 10^4$/μl	TP	5.9	g/dl
			Alb	2.9	g/dl
Serologic			BUN	14	mg/dl
CRP	5.4	mg/dl	Cr	0.5	mg/dl
			Na	138	mEq/l
			K	4.3	mEq/l
			Cl	100	mEq/l
			Ca	9	mg/dl

WBC white bold cell, RBC red blood cell, Hb hemoglobin, Ht hematocrit, MCV mean corpuscular volume, MCH mean corpuscular volume, MCHC mean corpuscular hemoglobin concentration, Plt platelets, AST aspartate aminotransferese, ALT alanine aminotransferase, ALP alkaline phosphatase, γ-GTP γ-glutamyltransferase, LDH lactate dehydrogenase, T-Bil total bilirubin, CPK ccreatine phosphokinase, TP total protein, Alb albumin, BUN blood urea nitrogen, Cr creatinine, Na sodium, K potassium, Cl chloride, Ca calcium, CRP C-reactive protein
