# Supplementary material for: Ileocecal ulcers accompanied by relapsing polychondritis: a case report
Source: Springerplus. 2014 Dec 7;3:714. doi: 10.1186/2193-1801-3-714 (PMC4320181; doi:10.1186/2193-1801-3-714)
Supplement: Supplementary file 7 — Authors’ original file for figure 7 [file 40064_2014_1482_MOESM7_ESM.pdf]

Table 2. Summary of the cases of MAGIC syndrome with GI lesions

**Case No.**	**Author**	**age/gender**	**clinical features**		
			chondritis	*oral ulcers*	*genital lcers*
1	Firestein GS et al.(6)	59,M	auricular chondritis	(+)	(+)
2	Imai H et al.(7)	39,F	auricular chondritis	(+)	(+)
3	Kotter I et al.(8)	59,M	auricular chondritis	(+)	(-)
4	Minami R et al.(9)	30,M	auricular chondritis,	(-)	(+)
5	Our case	30,F	auricular chondritis, nasal chondritis	(-)	(-)

ocular inflammation	*skin manifestation*	*gastrointestinal*
(-)	(-)	duodenal ulcer,intestinal fistula
keratoiritis	erythema nodosum	colitis
scleritis	(-)	aphthous colitis
scleritis	(-)	ileocecal ulcer
scleritis	(-)	ileocecal ulcer
